# A new method to suppress ghosting artifacts arising from long-T1 species in segmented inversion recovery (IR) sequences

**DOI:** 10.1186/1532-429X-13-S1-P9

**Published:** 2011-02-02

**Authors:** Nayla Chaptini, Wolfgang G Rehwald, Elizabeth R Jenista, Michele A Parker, Enn-Ling Chen, Raymond J Kim

**Affiliations:** 1 Duke University, Durham, NC, USA; 2 Siemens Healthcare and Duke University, Durham, NC, USA

## Objective

To evaluate a new long-T1 artifact suppression method and compare its effectiveness with previously reported techniques.

## Background

**Figure 1:**
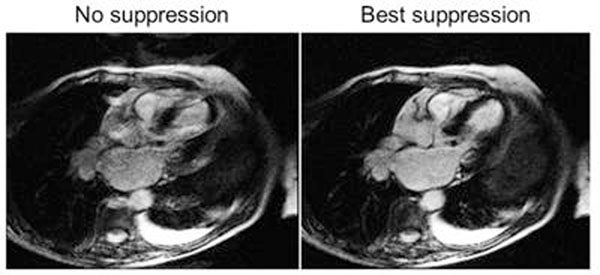
Example of long T1 ghosting artifact in a patient with a left pleural effusion.

Bright ghosting due to long-T1 species (e.g. cerebrospinal fluid, pericardial or pleural effusion, fig.1) often hampers the identification of infarcted myocardium on delayed enhancement CMR. The artifacts are caused by signal oscillations of the long-T1 species due to the periodic application of IR pulses. Previous methods use a dummy heartbeat (+HB, sequence run without data acquisition for leading heartbeat) or various pre-pulses (SR or IR). Both techniques may result in insufficient suppression or may be cumbersome to use. We developed a new suppression method utilizing saturation pulses (followed by “crusher” gradient pulses) after each readout (+Po) in combination with a dummy heartbeat. This method does not require user input. We tested it against four other suppression methods by quantifying the long-T1 ghosting seen in the respective images.

## Methods

**Figure 2:**
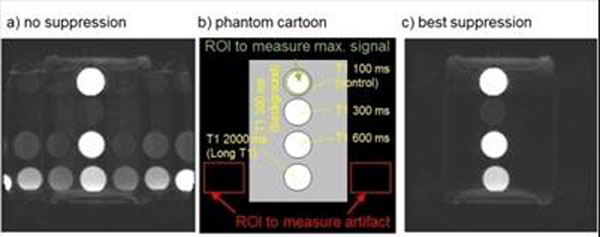
a) Example of long T1 ghosting artifacts using a phantom with different T1. b) Phantom diagram and annotations. c) Artifact suppression using our best suppression method.

A phantom containing five compartments with different T1-values (figure 2) was imaged (MAGNETOM Avanto, Siemens) with IR GRE and SSFP sequences with and without PSIR at heart rates (HR) 60 and 100 bpm. No suppression (NO) and five suppression methods were applied: 1) +HB, 2) -HB+Pre, 3) +HB+Pre, 4) -HB+Po, 5) +HB+Po. The inversion time (TI) was set to null the background at each HR. The images were quantified in ImageJ (NIH) as relative artifact signal by dividing the mean signal in the ghosting region of interest (ROI1) by the mean signal of the short T1 (brightest, control) compartment (ROI2, fig.2b). Additionally, artifacts were visually scored by three blinded readers, ordering them by quality (1 worst, 6 best). Signal-to-noise ratio (SNR) was measured in ROI2.

## Results

Table demonstrates a significantly lower relative artifact signal of method 5 compared to all other suppression techniques (p<0.05), for GRE and SSFP with and without PSIR, and independent of HR. Qualitative analysis the techniques from best to worst as 5, 3, 1, 2, 4, NO, where again method 5 performed best. SNR in control region ROI2 for method 5 was not significantly different from that of method 2 (p<0.05).

## Conclusions

The new post-suppression method (5) consistently showed the best ghosting suppression by qualitative and quantitative tests in both GRE and SSFP sequences, while preserving SNR. Due to its improved artifact suppression, simple implementation and heart rate independence, this method is currently used clinically at our site for delayed enhancement imaging.

**Table 1:** Artifact to maximal signal ratios (x100) for suppression method imaging type and heart rate.  Underlined numbers have the least artifact to signal ratio

Artifact-Signal Ratio (x 100)	GRE				SSFP			
	Magnitude		PSIR		Magnitude		PSIR	

	HR60	HR100	HR60	HR100	HR60	HR100	HR60	HR100

No Suppression	13.5	17.4	88.3	97.1	14.2	19	54	53.4
Suppression 1	2.8	9.3	10.2	54.6	4.2	9.5	25.8	44.8
Suppression 2	3.9	8.4	17.8	57.1	3.2	6.6	20.2	34
Suppression 3	2.8	3.2	10.5	13.8	1.2	3.4	3.4	20.5
Suppression 4	3.2	3.9	4.7	11.9	2.5	10.2	1.5	24.7
Suppression 5	2.2	2.2	0.7	1	0.6	0.6	0.5	0.1

